# Assessment of satisfaction, impact and emotional stress in elderly diabetics without satisfactory control of disease

**DOI:** 10.1186/1758-5996-7-S1-A171

**Published:** 2015-11-11

**Authors:** Rafael Vaz Machry, Luthiele da Silva Vasconcellos, Cibelle de Abreu Evaldt, Rafaela Ramos Nunes, Henrique Umpierre Pedroso, Thaymê Luísa de Souza Pires, Raquel Ferreira, Eduardo Bardou Yunes Filho, Paloma Dias da Cruz, Ticiana da Costa Rodrigues

**Affiliations:** 1 UFRGS, Porto Alegre, Brazil

## Background

Psychological stress is common in relation to the care needed for patients with chronic diseases. In addition, diabetes (DM) can present major impact on patients' lives and the degree of satisfaction with the established treatments. Patients without adequate glycemic control may be exposed to greater stress.

## Objectives

To assess the impact and the degree of satisfaction in the quality of life of patients with psychological stress related to DM in elderly patients without adequate glycemic control.

## Materials and methods

We performed a historical case-control study of diabetic patients in the Endocrinology Department of a Brazilian tertiary hospital who were treated between June and December 2014. We include patients over 60 yrs. of age, of both sexes, with HbA1c ≥ 8.5% using oral hypoglycemic agents and insulin. Patients were submitted to BPAID (Problems Ares in Diabetes – Brazil) and DQOL (Diabetes Quality of Life) questionnaires, both validated in Portuguese. The first evaluates emotional stress related to DM in 20 questions. On the second, we use the variables “impact” and “satisfaction” to evaluate quality of life. There were 33 issues. We divided the patients into two groups, according the mean of score BPAID (39.45). There are no pre-defined cutoff point for this BPAID questionnaire. The higher the final score, the greater the stress related to DM.

## Results

Forty-five patients were included. There were no differences between the groups for age, gender, education, race, religion, smoking and alcohol. There was no difference in HbA1c (10.75% vs. 9.98%, p=0.09) in the moment of interview. However, the HbA1c levels was greater in the year preceding study in patients with the worst stress scores (10.87% vs. 9.31%, p=0.023). Number of medicines used, dose of Insulin UI/kg, number of daily insulin injections or use of Regular insulin, weight, presence of hypoglycemia, blood pressure levels, or presence of chronic complications associated were not different. Patients with higher levels of stress presented greater negative impact of diabetes on quality of life compared to patients with lower levels of stress (p<0.001). Results were similar with respect to the degree of satisfaction (p<0.001).

## Conclusion

Emotional stress associated with DM can be related with worse quality of life in elderly diabetics and chronic glycemic uncontrol. Differences between social characteristics or relating to the treatment of DM did not differ between groups.

